# Genome-Wide Tissue-Specific Gene Expression, Co-expression and Regulation of Co-expressed Genes in Adult Nematode *Ascaris suum*

**DOI:** 10.1371/journal.pntd.0002678

**Published:** 2014-02-06

**Authors:** Bruce A. Rosa, Douglas P. Jasmer, Makedonka Mitreva

**Affiliations:** 1 The Genome Institute, Washington University School of Medicine, St. Louis, Missouri, United States of America; 2 Department of Veterinary Microbiology and Pathology, Washington State University, Pullman, Washington, United States of America; 3 Department of Medicine, Division of Infectious Diseases, Washington University School of Medicine, St. Louis, Missouri, United States of America; 4 Department of Genetics, Washington University School of Medicine, St. Louis, Missouri, United States of America; University of Melbourne, Australia

## Abstract

**Background:**

*Caenorhabditis elegans* has traditionally been used as a model for studying nematode biology, but its small size limits the ability for researchers to perform some experiments such as high-throughput tissue-specific gene expression studies. However, the dissection of individual tissues is possible in the parasitic nematode *Ascaris suum* due to its relatively large size. Here, we take advantage of the recent genome sequencing of *Ascaris suum* and the ability to physically dissect its separate tissues to produce a wide-scale tissue-specific nematode RNA-seq datasets, including data on three non-reproductive tissues (head, pharynx, and intestine) in both male and female worms, as well as four reproductive tissues (testis, seminal vesicle, ovary, and uterus). We obtained fundamental information about the biology of diverse cell types and potential interactions among tissues within this multicellular organism.

**Methodology/Principal Findings:**

Overexpression and functional enrichment analyses identified many putative biological functions enriched in each tissue studied, including functions which have not been previously studied in detail in nematodes. Putative tissue-specific transcriptional factors and corresponding binding motifs that regulate expression in each tissue were identified, including the intestine-enriched ELT-2 motif/transcription factor previously described in nematode intestines. Constitutively expressed and novel genes were also characterized, with the largest number of novel genes found to be overexpressed in the testis. Finally, a putative acetylcholine-mediated transcriptional network connecting biological activity in the head to the male reproductive system is described using co-expression networks, along with a similar ecdysone-mediated system in the female.

**Conclusions/Significance:**

The expression profiles, co-expression networks and co-expression regulation of the 10 tissues studied and the tissue-specific analysis presented here are a valuable resource for studying tissue-specific biological functions in nematodes.

## Author Summary

Tissue-specific gene expression provides fundamental information about the biology of diverse cell types within an organism and interactions among tissues within multicellular organisms. However, such studies are experimentally challenging in smaller organisms such as many nematodes species, including the species (*Caenorhabditis elegans*) that is widely used in biomedical research. *Ascaris suum* (the large roundworm of swine), however, is of particular interest as a model nematode because it is large enough to allow for the dissection of individual tissues, and equally important because it is closely related to *A. lumbricoides*, which infects ∼1 billion people worldwide. Here, we build significantly on the previous tissue-specific gene expression research in *A. suum* by producing the first nematode RNA-seq dataset that spans multiple specific tissues, including three non-reproductive and two reproductive tissues in both male and female *A. suum* worms. This analysis provides significant details on the biological functions occurring within each of these tissues, which has not been previously explored. It also provides insight into specific gene regulation pathways active in each of the tissues, which have broad applicability across other nematodes, including both non-parasitic and parasitic species.

## Introduction

Gene expression profiling is fundamental to understanding organismal biology, development and underlying functions at a specific time or under specific conditions. Tissue-specific gene expression provides fundamental information about the biology of diverse cell types within an organism and interactions among tissues within multicellular organisms. Molecular knowledge based on stage- and/or tissue-specific gene expression profiles in model organisms is explored to understand many aspects of complex diseases, and in parasitic helminths is explored to identify the properties/functions of tissues that may serve as targets for treatments and control measures. However, such studies (especially high-throughput tissue-specific gene expression studies) are experimentally challenging in smaller organisms, such as many nematodes species [1].

The phylum Nematoda is composed of the most abundant and diverse species of all animal phyla, with an estimated million species that are found in almost every environment including extremes such as hot springs and polar ice [2]. Members of this phylum are free-living or parasitic, and include one of the most well-studied model organisms, *Caenorhabditis elegans*. Of the ∼28,000 described nematode species, ∼16,000 are parasitic [3]. Infections by parasitic nematodes cause extensive suffering in humans, animals, and plants, as well as major losses in agricultural production due to disease and the cost of implementing control programs [4]. Calculations of the aggregate burden of human nematode diseases in Disability Adjusted Life Years (DALYs) indicate a tremendous global impact of these pathogens [5]. Research progress on anthelmintic discovery and immunological control of parasitic nematode infections has been impeded by the biological complexity of nematodes and their interactions with the host.

Extensive and high-quality genomic databases are available for *C. elegans* [6] and are also emerging for parasitic nematodes [7], providing a welcome infusion of information that opens valuable new avenues for progress in nematode research. This information has been used to produce stage-specific high-throughput gene expression experiments for nematode species using conventional expressed sequence tags (ESTs), next-generation sequencing (454/Roche, Illumina) or microarrays (e.g. [8], [9], [10], [11]), providing many novel insights into nematode biology. The elucidation of gene repertoires expressed by specific tissues of nematodes can further facilitate the development of broader and deeper insights into individual tissue functions, with applications to parasitic and non-parasitic nematodes alike. At the comparative level, information of this kind will aid in understanding of both conserved and divergent aspects of nematode biology, while also enhancing the value of model organisms, such as *C. elegans*, in biomedical research. However, due to the small size of nematodes, it is not possible to accurately dissect enough tissue in most of these species to run high-throughput gene expression, proteomics or cellular biochemical experiments at the individual, isolated tissue level. Bioinformatic-based predictions of tissue-specific high-throughput gene expression have been inferred based on whole-organism, stage-specific *C. elegans* microarray data [1], but these computational tissue-specific expression predictions still await experimental confirmation and are not useful in identifying genes relevant to host-parasite interactions, since *C. elegans* is a free-living species [12].

**Figure 1:**
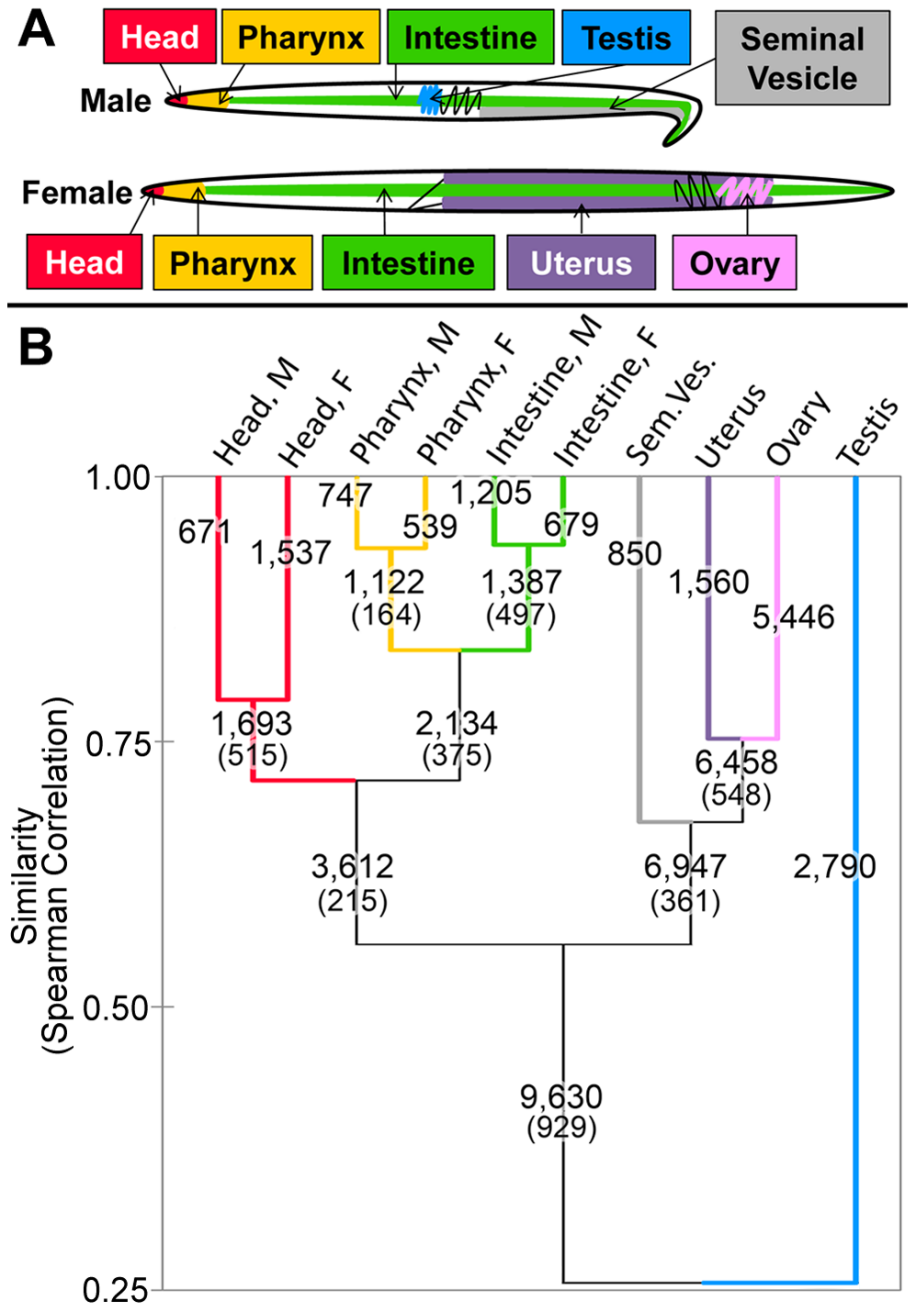
(A) Simplified diagram of the tissues selected for deep RNA sequencing in male and female adult *A. suum* worms. (B) Hierarchical clustering of *A. suum* RNA-seq samples based on gene expression data across all expressed genes. Numbers above lines represent the number of genes overexpressed in each branch of the clustering and numbers below lines in parentheses represent the number of genes overexpressed in both of its child branches.

In this context, *Ascaris suum* (the large roundworm of swine) is of particular interest as a model parasitic nematode. The *A. suum* genome has recently been sequenced [13], and this parasite serves as a research model for its close relative, *A. lumbricoides*, which is responsible for widespread disease infecting over one billion people worldwide [14]. The large size of *A. suum* relative to other nematodes (adults can reach up to 40 cm in length) allows for accurate dissection of individual tissues and organs that is not possible in smaller nematodes. Previous studies have analyzed expression from a single tissue in *A. suum* using conventional ESTs [15] and then multiple tissues using microarrays [16]. The multiple tissue study [16], while providing insight on many biological functions of tissues investigated, was based on ∼40,000 60-mer (40-k array) elements derived from genes predicted from low coverage of the *A. suum* genome, resulting in multiple elements representing a single gene or genes not being represented in the partial genome. Indeed, when the *A. suum* genome became available [13] the 40-k array elements was shown to cover just 58% of the predicted *A. suum* genes [16]. This limitation has made it challenging to identify exact genes contributing to the expression patterns, and did not take advantage of the wide range of functional information that can be annotated using full-length gene sequences, or the high expression-level accuracy that can be provided with RNA-seq analysis. Here, we build significantly on the previous tissue-specific gene expression research in *A. suum*, by producing the first nematode RNA-seq dataset that spans multiple specific tissues, including three non-reproductive and two reproductive tissues in both male and female *A. suum* worms (Fig. 1A). The analysis presented here provides additional annotation to the *A. suum* genome, through i) tissue-specific gene expression profiling, ii) detailed Gene Ontology-based functional enrichment for each tissue, iii) delineation of putative cis and trans regulatory elements involved in regulating expression in the specific tissues investigated and iv) co-expression networks, which identified putative genes that link molecular pathways across different tissues. The 10-tissue specific expression profiles (Supp. Table S1; Also available on www.nematode.net [7]) and the analysis presented here provide valuable resources for studying basic functional relationships in nematodes, including both non-parasitic and parasitic species.

## Methods

### Parasite material and RNA preparation

Adult worms were collected from infected pigs at an abattoir when being processed as part of the normal work of the abattoir. The fresh worm tissues, including three non-reproductive (head, pharynx, and intestine) tissues in both male and female worms, as well as two reproductive tissues per sex (testis, seminal vesicle, ovary and uterus) were dissected (Fig. 1) and snap frozen in liquid nitrogen for subsequent storage at −80°C. Note that while the term “tissue” is used to describe all of these samples for simplicity, they may be more accurately described as “organs” or “regions of the body” rather than pure tissues. For the non-reproductive “tissues”, the “head” is defined as the terminal anterior region of the worm anterior to the beginning of the muscular pharynx, the “pharynx” is defined as the anterior body region that extends from the anterior-most to posterior-most margins of the muscular pharynx, and the “intestine” is defined as posterior to the posterior-most margin of the muscular pharynx to the posterior-most limit of the intestine near the anus (female) or cloaca (male). In the male reproductive samples, the “testis” sample contained the entire male reproductive system distal to the seminal vesicle. The “seminal vesicle” itself is likely to contain sperm, spermatids and vesicle wall. For the female reproductive system, some eggs were present in the “uterus” tissue, and while they are relatively resistant to Trizol, some contribution of mRNA from eggs in the uterine preparation cannot be excluded. The “ovary” samples contained the female reproductive system distal to the oviduct.

Tissue homogenization and RNA extraction were performed using TRIzol (Invitrogen; according to the manufacturer's instruction) and a rotor/stator probe (Tissue Tearor Model 985270-395, BioSpec Products Inc), which was used to mix the samples for 15 second intervals until the samples were completely homogenized. The integrity and yield of the RNA was verified by the Bioanalyzer 2100 (Agilent Technologies, Cedar Creek, Texas). Total RNA was treated with Ambion Turbo DNase (Ambion/Applied Biosystems, Austin, TX), and 1 ug of the DNAse-treated total RNA went through polyA selection via the MicroPoly(A) Purist Kit according to the manufacturer's recommendations (Ambion/Applied Biosystems, Austin, TX). 1 ng of the mRNA isolated was used as the template for cDNA library construction using the Ovation RNA-Seq (version 2) kit according to the manufacturer's recommendations (NuGEN Technologies, Inc., San Carlos, CA). Whole-worm male and female samples were prepared using the same protocol.

### RNA-seq library construction and sequencing

Non-normalized cDNA was used to construct Multiplexed Illumina paired end small fragment libraries according to the manufacturer's recommendations (Illumina Inc, San Diego, CA), with the following exceptions: 1) 1 ug of cDNA was sheared using a Covaris S220 DNA Sonicator (Covaris, INC. Woburn, MA) to a size range between 200–400 bp. 2) Four rounds of PCR amplifications were performed to enrich for proper adapter ligated fragments and properly index the libraries. 3) The final size selection of the library was achieved by an AMPure paramagnetic bead cleanup (Agencourt, Beckman Coulter Genomics, Beverly, MA), targeting 300–500 bp. The concentration of the library was accurately determined through qPCR according to the manufacturer's protocol (Kapa Biosystems, Inc, Woburn, MA) to produce cluster counts appropriate for the Illumina GAIIx platform. Multiple libraries were pooled together and loaded into one lane of a HiSeq2000 version 3 flow cell. 2×101 bp read pairs (later clipped to 100 bp using Consensus Assessment of Sequence and Variation [CASAVA, version 1.8]) were generated for each sample, generating ∼2 Gb per sample. Whole-worm male and female samples were sequenced using the same protocol (SRA Accession numbers SRR851237, SRR851252, SRR851258 and SRR869505).

### Analytical processing of the reads and differential expression

Analytical processing of the Illumina 100 bp reads was performed using in-house scripts. DUST was used to filter out regions of low compositional complexity and to convert them into Ns [17]. An in-house script was used to remove Ns, which discards reads without at least 60 bases on non-N sequence. Sequences from host (pig genome; Sscrofa9.2, GCA_000003025.2 from GenBank [18]), bacteria (GBBCT from GenBank [18]), and an *A. suum* mitochondrial database were screened using the *A. suum* Illumina short-reads. The number of RNA-seq reads identified and mapped per tissue sample is listed in Table 1. Processed and raw paired-end RNA-seq datasets are deposited at SRA (Accession Numbers SRR85166, SRR85167, SRR851186-SRR851203, SRR851213, SRR851223-SRR851225, SRR851254-SRR851257, SRR851632-SRR851637, SRR851639-SRR851641, SRR851855-SRR851857, and SRR869476; http://www.ncbi.nlm.nih.gov/sra). Whole-worm male and female samples were processed using the same protocol (Supp. Fig. S1).

**Table 1:** RNA-seq statistics for tissues-specific samples.

Tissue (M = Male, F = Female)	Number of Mapped Reads (Million)		Pearson Correlation Between Replicates	# of Genes >50% Breadth of Coverage
	Rep. 1	Rep. 2		
M Head	11.8	9.1	0.94	10,015
F Head	9.0	10.4	0.96	11,709
M Pharynx	7.6	10.1	0.61	9,787
F Pharynx	8.9	7.1	0.80	9,612
M Intestine	11.5	16.6	0.96	9,563
F Intestine	14.3	10.6	1.00	8,436
Testis	6.2	8.0	0.91	10,757
Ovary	9.7	16.1	0.89	11,652
Seminal Ves.	9.8	8.1	0.91	9,976
Uterus	8.7	6.1	0.98	10,279
Total	199.4		0.90 (average)	16,854 (merged)

Gene expression for each sample was calculated by mapping the screened RNA-seq reads to the recently released *A. suum* genome [13] using Tophat [19] (version 1.3.1), and calculating depth and breadth of coverage using Refcov (version 0.3, http://gmt.genome.wustl.edu/genome.shipit/gmt-refcov/current). Gene expression values were normalized using the depth of coverage per million reads (DCPM) per sample [20]. Stage-specific over-expression and under-expression for each gene with at least 50% breadth of coverage across all of the tissues was tested using SAMSeq (v4.0, released 2011 [21]). Genes with less than 50% breadth of read coverage of the gene sequence across all samples were excluded from the analysis. This algorithm was chosen because (i) it has been designed for multi-class testing among RNA-seq datasets (i.e. allows for more than pair-wise comparisons simultaneously, and can identify over-expression in multiple tissues), (ii) it has been shown to have low bias and false discovery rates relative to other differential expression algorithms for other RNA-seq datasets [22], [23], [24], and (iii) it has demonstrated effectiveness in other studies [21], [25], [26], [27], [28]. This algorithm identified approximately 69% of the expressed genes as being over-expressed in at least one of the tissues (with p≤0.05 confidence and a false discovery rate of 0.8%). Tissue-overexpression profiles for every gene were generated based on these results (Supp. Table S1; Also available on www.nematode.net [7]). In this context, the term “overexpression” is used to denote significantly higher expression for a gene in any given tissue, relative to the other tissues according to the test described above.

Gene expression levels (DCPM) for the two replicates in every tissue were averaged, and the samples were clustered based on their expression across all genes using hierarchical agglomerative clustering (with “unweighted pair group method with arithmetic mean”, and “Spearman correlation coefficient similarities” settings in XLSTAT-Pro version 2012.6.02, Addinsoft, Inc., Brooklyn, NY, USA; Fig. 1B).

### Functional annotation and enrichment

**Figure 2:**
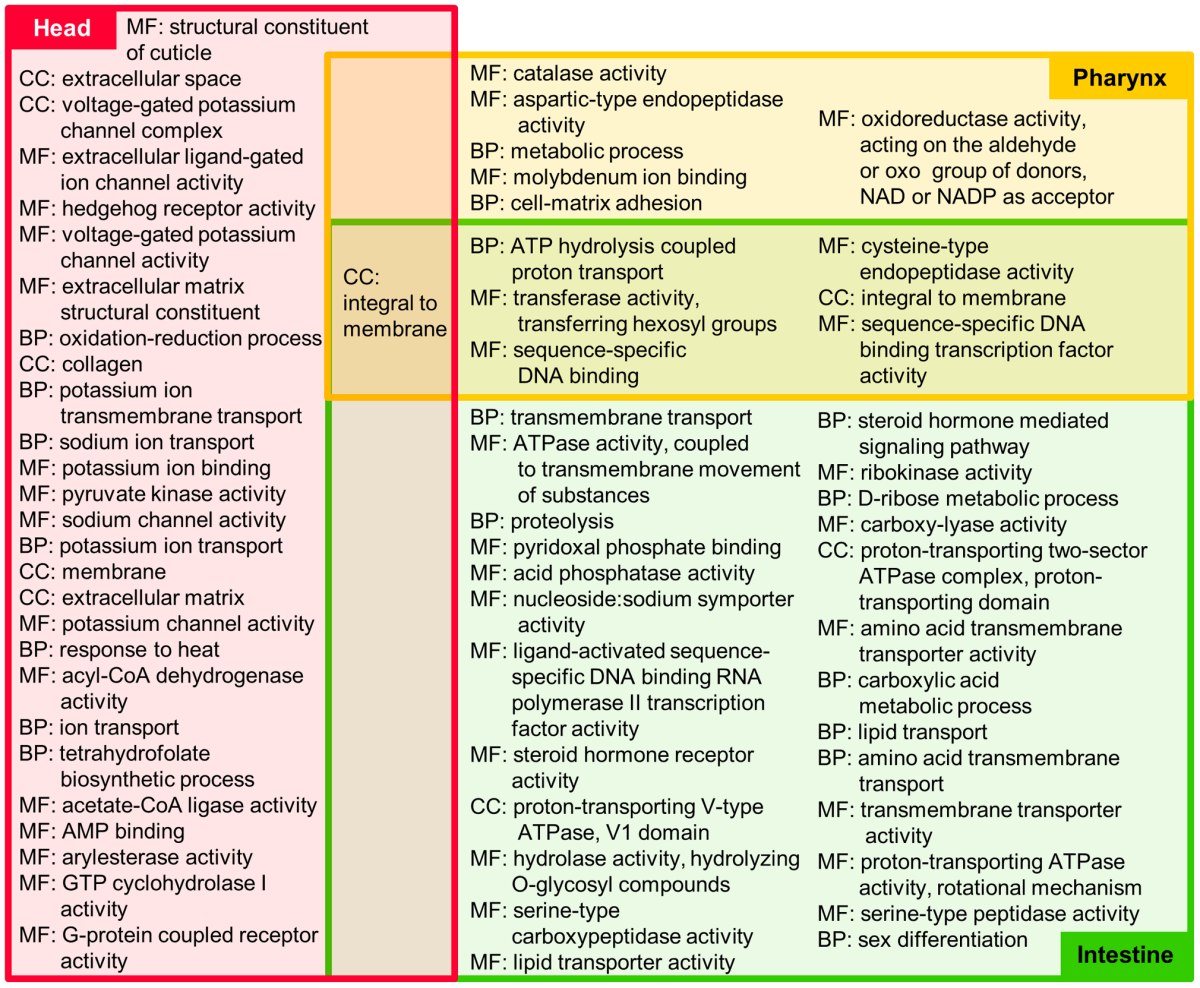
Gene Ontology (GO) terms significantly (p≤0.01, FDR corrected) enriched among genes overexpressed in non-reproductive tissues according to FUNC. GO root terms were abbreviated (MF = Molecular Function, BP = Biological Process, CC = Cellular component) and terms are sorted according to descending enrichment significance.

**Figure 3:**
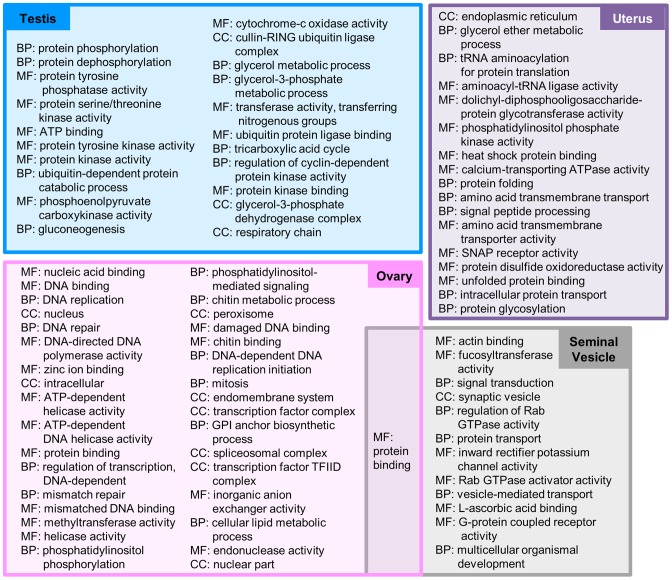
Gene Ontology (GO) terms significantly (p≤0.01, FDR corrected) enriched among genes overexpressed in reproductive tissues according to FUNC. GO root terms were abbreviated (MF = Molecular Function, BP = Biological Process, CC = Cellular component), and terms are sorted according to descending enrichment significance.

Interproscan [29], [30] was used to determine associations of genes to Gene Ontology (GO) terms [31]. Interproscan also identified predicted Interpro domains found in each gene. In addition, predicted proteins were searched against the KEGG database [32] using KAAS [33]. Proteins with signal peptides and transmembrane were identified using the Phobius [34] web server, and non-classical secretion was predicted using SecretomeP 1.0 [35]. FUNC [36] (which considers the hierarchical structure of GO) was used to determine significant functional enrichment among the genes overexpressed in each tissue, with a p≤0.01 significance threshold (after FDR population correction; Figs. 2 and 3, Supp. Table S2). For the non-reproductive tissues, overexpressed genes from both the male and female organs were pooled for the enrichment analysis. Interpro domain enrichment was determined using a non-parametric binomial distribution test with a p≤10^−5^ significance threshold (after FDR population correction). Only Interpro domains found in at least five predicted proteins were considered for enrichment testing (Supp. Figs. S2 and S3).

### Comparisons to *C. elegans*

Reciprocal best hits between predicted *A. suum* proteins based on the current version of the genome [13] and predicted *C. elegans* proteins from WormBase WS230 [37] were identified using WU-BLAST with a minimum bit score of 60 for each identified pair (using the parameters “hitdist = 40 wordmask = seg postsw”) (Table 2). All available isoforms of the proteins were used as input in this comparison. Within the sets of *A. suum* genes overexpressed in each tissue, enrichment of genes with reciprocal best hits to *C. elegans* was tested using a non-parametric binomial distribution test with p≤0.05 significance cutoff (after FDR population correction for the total number of tissues).

**Table 2:** Enrichment of reciprocal *C. elegans BLAST hits* among tissue-overexpressed genes.

Tissue	Number of Genes Overexpressed	Fraction of overexpressed predicted proteins with high-similarity reciprocal *C. elegans* BLAST hits (%)*	P-value, enrichment of genes with high-similarity reciprocal *C. elegans hits*
Head	1,693	40.8%	2.0×10^−5^
Pharynx	1,122	34.9%	0.57
Intestine	1,387	39.4%	0.0024
Testis	2,790	18.8%	1
Ovary	5,446	43.9%	<1×10^−12^
Seminal Vesicle	850	43.9%	1.3×10^−5^
Uterus	1,560	45.6%	1.2×10^−12^

* 34.9% of all genes expressed in this study had reciprocal *C. elegans* BLAST hits.

### Binding motif enrichment and annotation

**Figure 4:**
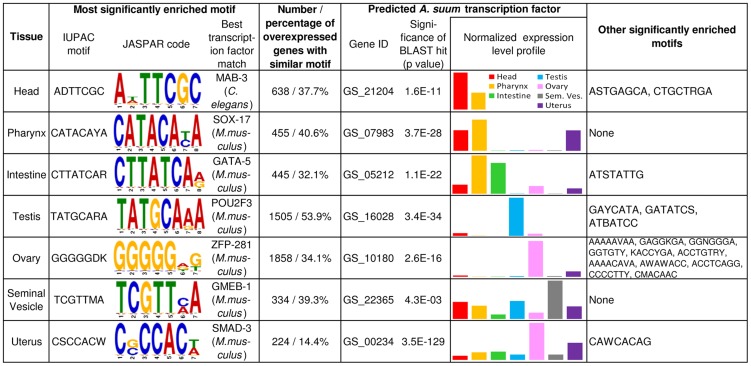
Transcription factor binding motif enrichment in the 5′ untranslated regions (UTRs) of genes overexpressed in specific tissues.

The identification of genes that are overexpressed in individual *A. suum* tissues facilitated the analysis of potential cis and trans regulatory elements responsible for this differential expression. 2000 bp upstream untranslated regions (UTRs) were extracted for each gene based on the *A. suum* genome annotation [13]. The 5′ end of 725 gene sequences (4% of the gene set) was less than 2000 bp from the end of a contig; these genes were not included the in motif enrichment testing. Motif enrichment was performed using a discriminative motif analysis algorithm (DREME [38], using an 8-nucleotide sequence search), where the 5′ UTRs of the genes overexpressed in a tissue were compared to the 5′ UTRs of the expressed genes not overexpressed in that tissue, in order to determine over-represented enriched motifs. FIMO [39] was used to calculate the coordinates of motifs similar to the enriched motifs among all genes, and potential transcription factors binding the discovered motifs were identified using Tomtom [40] (where transcription factors from the JASPAR CORE nematoda and vertebrata motif databases [41], as well as the UniProbe motif database [42] were considered for annotation). It should be noted that the Tomtom transcription factor binding site [7], [40] databases used to annotate the motifs described below (including the JASPAR vertebrate and nematode database [41] as well as the UniProbe [42]) contained only five nematode sequences, and hundreds of vertebrate sequences (primarily from *Mus musculus*), so many of the best-hit motif annotations described below are based on transcription factor data from mice due to bias in the best databases available. BLASTP [43] was used to identify potential orthologs of these transcription factors in the *A. suum genome*. The top five BLAST hits were considered for selection as the probable tissue-specific transcription factor, and the optimal target was chosen from these five based on gene annotation as well as on the tissue-specific gene expression profile for each potential TF (Fig. 4; Supp. Table S3). Supp. Table S4 contains a key for the base ambiguity among the motifs shown in Fig. 4 [44].

### Constitutively expressed and novel genes

**Figure 5:**
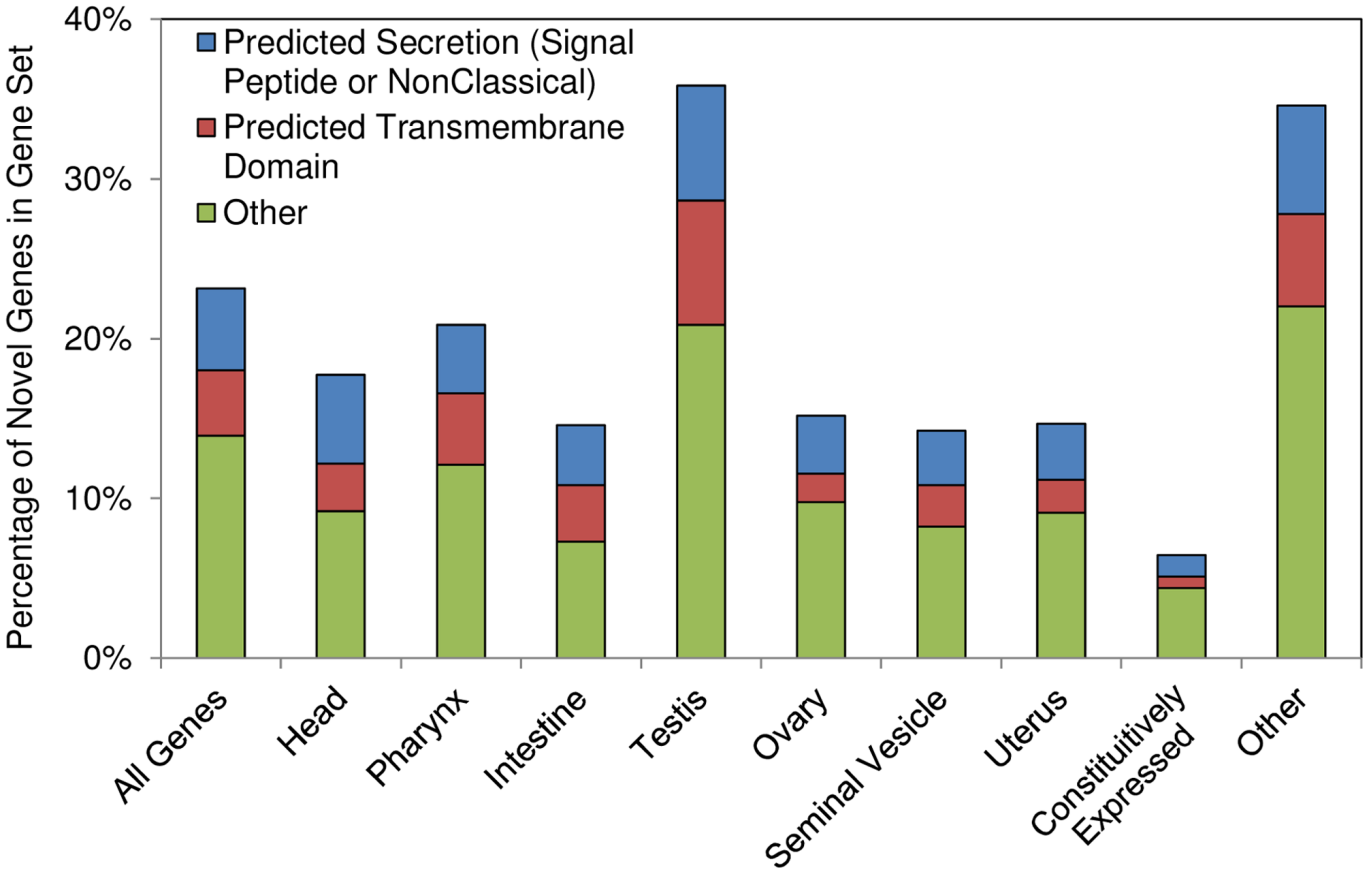
Novel gene distribution among all genes, genes overexpressed in each tissue, and constitutively expressed genes.

Constitutively expressed genes were identified based on the criteria that they were not significantly differentially expressed among tissues, and the minimum expression level in every tissue was greater than the median expression level for the entire dataset (0.26 DCPM), and novel genes were identified based on the the criteria that there was no annotation from best hit in the NCBI's NR database (provided in the original genome publication [13]), and no Interpro, GO or KEGG annotations (Figure 5).

### Gene co-expression network construction

**Figure 6:**
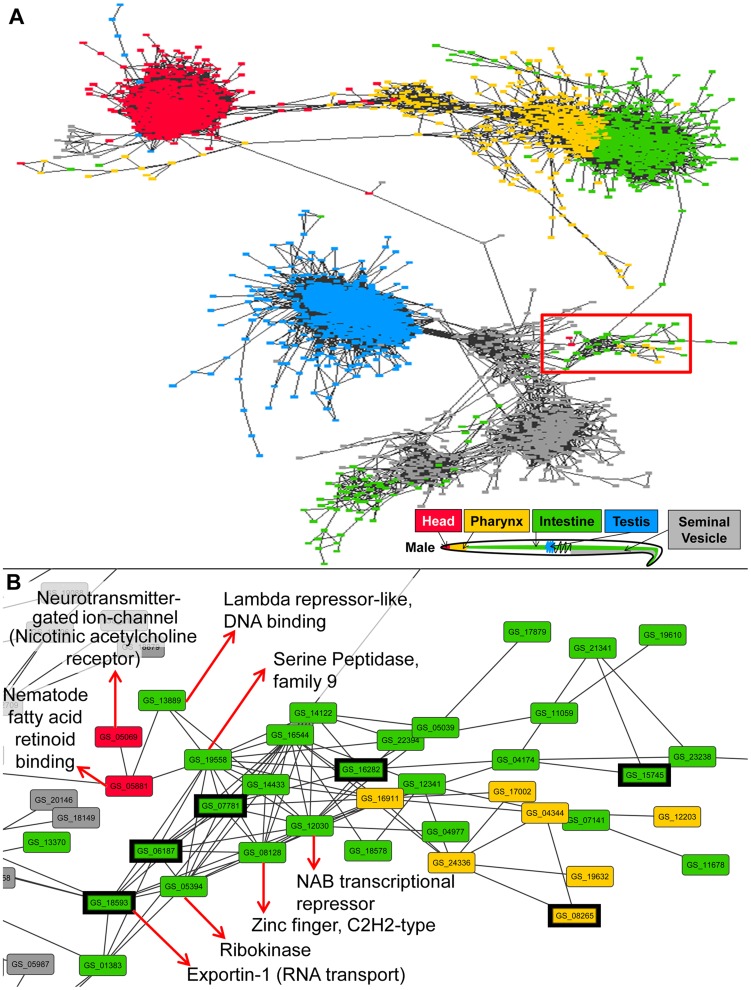
Gene co-expression network for genes overexpressed in at least one male tissue. (A) The complete network. (B) The subnetwork highlighted in the red box in A. Important functional genes are labelled based on their annotated Interpro domains, and genes annotated with the Gene Ontology term “Regulation of transcription, DNA dependent” (GO:0006355) are highlighted with a thick black border.

**Figure 7:**
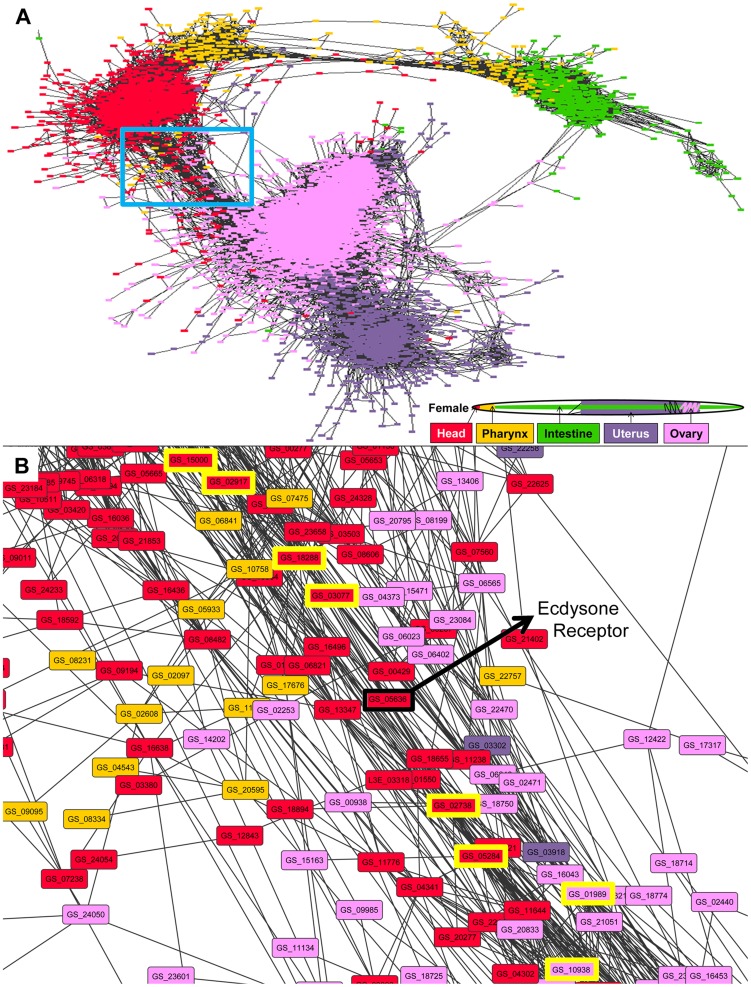
Gene co-expression network for genes overexpressed in at least one female tissue. (A) The complete network. (B) The subnetwork highlighted in the red box in A. The Ecdysone receptor gene is labelled, and the first-neighbors (i.e., highly correlated) genes to this gene are highlighted with a thick yellow border.

Male and female gene co-expression networks were constructed to further explore the tissue-specific gene expression data. For the male network (Fig. 6A), all genes overexpressed in at least one male tissue were considered for the network. For these genes, the Pearson correlation (based on the expression values across both replicates in all 10 tissues) between all gene pairs was calculated, and every gene pair with a correlation ≥0.90 was connected with an edge using Cytoscape software (version 3.0) [45]. It should be noted that a previous study showed that a Pearson correlation-based gene co-expression network of over 22,000 genes constructed with only 14 samples was sufficient to identify the same functional modules as a much larger dataset, so the 20 samples used here are thought to be sufficient to find biologically meaningful modules and subnetworks [46]. The male network contained 4,784 genes with 1,387,028 edges. The default “prefuse force-directed” layout was used with the “spring length” variable set to 100 in order to avoid overlapping of unconnected subnetworks. The positions of four nodes in the long vertical bridges of this network were manually repositioned in order to better display the connectivity without overlaps. Genes were colored according to the tissue in which they were overexpressed; if a gene was overexpressed in more than one tissue, the tissue with the highest expression level was chosen for the color coding. The same approach and settings were used to construct the female gene co-expression network (Fig. 7A), and this network contained 7,741 genes with 1,188,989 edges. High-resolution images of these networks as well as the Cytoscape network files are available on www.nematode.net [7].

## Results/Discussion

### RNA-seq analysis of 10 tissues dissected from adult male and female *A. suum*

RNA-seq analysis was performed in duplicate on three non-reproductive (head, pharynx, and intestine) tissues in both male and female worms, as well as two reproductive tissues per sex (testis, seminal vesicle, ovary and uterus) (Fig. 1A; Methods). Across the 20 RNA-seq samples (2 replicates from 10 different tissues), 348 million reads were generated, and 199 million reads mapped to the 18,542 genes of the *A. suum* genome [13]. The number of reads mapped in individual tissues ranged from 6 million (in the second uterine replicate) to 16 million (in the second male intestinal replicate; Table 1). The average Pearson correlation for expression values of all expressed genes between replicates was 0.90, while the average correlation between samples from different tissues was 0.24 (based on all pair-wise comparisons between tissues). The lowest correlation among replicates was between the replicates of the female pharynx (0.61), and may reflect the relative difficulty of the dissection procedure for this particular tissue (see Methods). A total of 16,854 genes (91% of the complete *A. suum* geneset) had ≥50% breadth of coverage (ie, ≥50% of the gene sequence was covered with at least one read from any of the samples), and this final set of expressed genes was used in the subsequent differential expression analysis, which identified 11,690 genes (63% of all expressed genes) as being significantly overexpressed in at least one tissue. The male and female samples had relatively consistent gene expression profiles in the head, pharynx and intestinal tissues (Fig. 1B). The tissue with the most distinct gene expression profiles was the testis, which had a low similarity (Spearman correlation of 0.26) compared to the other tissues. However, the similarity between the two testis replicates based on the same statistics was over 0.90 (data not shown), indicating that this large difference is not due to inter-replicate variability.

Unlike the previous microarray-based study of *A. suum* tissues [16], genes with high similarity to *C. elegans* genes were found to be more enriched among overexpressed genes in all tissues except for the testis and pharynx (Table 2). In the previous study, similarity was measured by the identification of PANTHER domains among the genes, which may have biased the identification towards genes with known functions rather than genes with similarity to other species. Also, unlike the previous microarray-based study of *A. suum* tissue-specific expression which found substantial differences between genders in terms of expression profiles in non-reproductive tissues (particularly the intestine) [16], we observed strong agreement between the gene expression profiles for the male and female intestine and pharynx (Spearman r = 0.93 and 0.94, respectively), but observed a higher disparity for the head (r = 0.79). While this difference may be biological in nature, it may be accounted for by the higher accuracy of the expression data here, provided both by RNA-seq (as opposed to microarray) and the higher-quality gene set provided by the recent genome publication (which was not available when the microarray study was performed [16]). The current study focuses primarily on tissue-specific differences among these tissues rather than on the gender differences.

Separate whole-worm male and female *A. suum* RNA-seq samples were also generated as a comparison to these tissue-specific samples. In the whole-worm male sample (Supp. Fig. S1A), 15,604 genes were detected (*see* methods for criteria), and in the combined tissue-specific male samples, 15,941 genes were detected, including 863 which were not detected in the whole-worm samples. Among those 863, more than half (52%) had their highest expression in either the head or pharynx (compared to 34% in the entire tissue dataset). Likewise, in the female comparison (Supp. Fig. S1B), 45% of the 1,655 genes detected only in the tissue-specific dataset were most highly expressed in the head or pharynx, compared to 31% across all of the genes expressed in the tissue samples (Supp. Table S1). Thus, the whole-worm samples more often failed to capture the expression of genes which are most actively expressed in the head and pharynx, which highlights the importance of the production of these tissue-specific datasets.

### Functional enrichment in non-reproductive tissues

Interproscan [29], [30] was used to determine associations of genes to Gene Ontology (GO) terms [31], and FUNC [36] (which considers the hierarchical structure of GO) was used to determine significant functional enrichment among the genes overexpressed in each tissue, with a p≤0.01 significance threshold (after FDR population correction; Figs. 2 and 3, Supp. Table S2). In the context of this study, “overexpression” denotes significantly higher expression in a given tissue relative to the other tissues, and genes may be overexpressed in more than one tissue or no tissues (see Methods, “Analytical processing of the reads and differential expression”).

The most enriched term in the head of *A. suum* (including the circular three-lipped mouth, the outer cuticle layer, amphids, some muscle tissue, and internal structures consisting primarily of neurons) was “structural constituent of cuticle” (GO:0042302). This term was identified as being enriched in the head of *A. suum* in the previous microarray study [16], and was enriched only in the head-overexpressed genes in this study, which could be expected since most of the other internal tissues are not cuticle-lined, or are only partially lined with cuticle (e.g. pharynx or the distal posterior part of the intestine-anus). Additionally, many of the GO terms exclusive to the head (Fig. 2) are linked to neuronal activity, including ten terms related to ion channel/transport activity (related to synaptic transmission and action potential depolarization in neurons [47], [48]), which were not previously identified [16]. “Arylesterase activity” (GO:0004064) was also enriched, which is of interest because arylesterase is negatively correlated with inflammation in mammals [49], and was found to be significantly decreased in the serum of rats infected with the nematode *N. brasiliensis* [50]. This presents a possible mechanism by which *A. suum* achieves its anti-inflammatory properties inside the host, and genes annotated with this term may be of interest for future immunological studies [51].

Most research performed on the nematode pharynx has focused on the anatomy, development and neuronal connectivity of the pharynx, primarily because it is an excellent model for organogenesis [52], [53]. In *C. elegans*, pharyngeal secretions are thought to be involved in digestion, but the nature of those secretions is largely unknown [52]. The previous microarray-based study found no significant functional enrichment for the pharynx [16], but here we have identified twelve enriched terms. This difference is likely due to an improved dataset resulting in more comprehensive coverage of the genome, as well as the recent improvement of the genome itself. Five child terms of “catalytic activity” (GO:0003824) were found to be significantly enriched in the pharynx (including two which were also enriched in the intestine). Necepsins have nematode-specific characteristics and are able to hydrolyse host proteins including hemoglobin and serum proteins [54], and eight out of the nine genes annotated as necepsins (according to NCBI RefSeq database search results provided in the genome publication [13], [55]) were also annotated with “aspartic-type endopeptidase activity” (GO:0004190, enriched in the pharynx; p = 8×10^−4^).

Nearly half (46%) of the *A. suum* intestinal transcripts conserved with *H. contortus* and *C. elegans* in a previous study [56] were identified in our *A. suum* intestinal genes, representing 395 unique intestinal genes (enriched for overexpression in the intestine, p<10^−10^). Here, a total of 31 GO terms were significantly enriched in the intestinal tissue, including nine ‘Molecular Function’ child terms of “hydrolase activity” (GO:0016787) (including “cysteine-type endopeptidase activity”, a category previously identified in the *A. suum* intestine [16]) and eleven terms related to transport of protons, lipids and amino acids.

### Functional enrichment in reproductive tissues

The testis in *A. suum* is the best characterized of the four reproductive tissues analyzed in this study. A previous study focused on the functional activity in the *A. suum* testis identified genes with phosphatase and kinase activity as being particularly overrepresented in this tissue [57]. It is speculated that this catalytic activity relates to the discarding of protein synthesis-related machinery in sperm and an upregulation of genes required for pseudopod extension and sperm cell motility [57], and proteins expressed specifically in the testis that are lost during chromosome diminution were found to be enriched for these functions [57], [58]. Although not found in the previous microarray-based study [16], the presence of high phosphatase activity in the testis is supported here by the enrichment of the MF GO term “protein tyrosine phosphatase activity” (GO:0004725), and the presence of high kinase activity is supported by the enrichment of six different terms describing kinase activity. Five *A. suum* major-sperm protein (MSP) domain-containing proteins were previously found to be active in the testis [57]; The *A. suum* genes with the highest sequence similarity to each of these MSP genes was found via a BLAST search [43], and all five were over-expressed in the testis in this analysis (Supp. Table S5). Also, previously, a serine protease inhibitor expressed in the *A. suum* testis (As_SRP-1) was found to be critical for cytoskeleton assembly and motility [59]; The *A. suum* gene with the highest similarity to *As_SRP-1* (*GS_04617*) was very highly overexpressed in the testis (with the 6^th^ highest average expression value of all the genes in the testis).

Unlike the testis, the broad molecular activity in the ovary, seminal vesicle and uterus of *A. suum* have not been previously studied outside of the previous microarray study [16]. However, a number of studies have identified many genes responsible for different stages of embryo and oocyte development in the ovary of *C. elegans* [60], [61], including a study which estimated that more than 2,600 genes are responsible for these processes alone [62]. Here, more genes were overexpressed in the *A. suum* ovary than in any other tissue (5,446; Fig. 1B). The top five enriched GO terms (and fourteen total terms) were directly related to DNA binding and replication, including “mitosis” (GO:0007067; Fig. 3), consistent with an RNA-seq dataset produced from the *A. suum* genome publication [13], and demonstrating that our approach is identifying the expected biological functions in the ovary. Two BP GO terms related to phosphatidylinositol signalling were also enriched in the ovary, which supports previous literature suggesting that at least one of these signalling pathways (the *ppk-1* pathway) is necessary for ovulation in *C. elegans* [63]. Also, two terms related to chitin binding were found to be enriched among ovary-overexpressed genes, consistent with findings in the previous microarray study [16].

Like with the ovary, very little is known about the specific molecular functional activity of the seminal vesicle in *A. suum* [16], [64], and genes overexpressed in this tissue (and the uterus) were enriched for sharing high sequence similarity (based on reciprocal BLAST hits) to *C. elegans* (p = 1×10^−5^). In *C. elegans*, seminal fluid has been shown to modulate sperm function, promote sperm viability and initiate physiological changes in the female uterus [65]. Actin and cytoskeleton activity have been shown to be critically important for nematode sperm motility and activation [66], consequently it is possible that the high enrichment of the MF GO terms “protein binding” (GO:0005515) and “actin binding” (GO:0003779) in the seminal vesicle is due to the overexpression of several genes responsible for binding spermatids (Fig. 3). In addition, “fucosyltransferase activity” (GO:0008417) was found to be enriched in the seminal vesicle, a function which has also been found in the seminal fluid of mammals and implicated in fertility via the removal of fertility-inhibiting fucose-containing molecules on the sperm surface [67], but this observation has not been previously reported in the literature for nematodes.

The *A. suum* uterus is the site of fertilization and egg development, and as with the ovary and seminal vesicle, *A. suum*-specific studies of the uterus have focused on morphology rather than detailed functional analysis [68], [69]. Genes overexpressed in the *A. suum* uterus were enriched for sharing high sequence similarity with *C. elegans* (1×10^−12^), but only limited knowledge of the biological pathways in the mature *C. elegans* uterus is available, as most research has focused on uterine developmental pathways rather than functional activity in the adult uterus [70], [71]. Here, we have identified a range of molecular functions associated with the *A. suum* uterus (Fig. 3), including four child terms of “protein binding” (GO:0005515) and four child terms of “catalytic activity” (GO:0003824).

Highly and significantly enriched (p≤10^−5^) Interpro domains among genes in each of the tissues studied are shown in Supporting Figures S2 and S3. These domains are consistent with the GO term enrichment results, since they were both based on Interproscan identifications [72].

### Tissue-specific transcription factor binding site enrichment

The identification of genes that are preferentially or exclusively expressed in individual *A. suum* tissues facilitated the analysis of potential cis and trans regulatory elements responsible for this differential expression. The sequences upstream of the first base of the gene models (up to 2000 bp) were examined for potential transcription factor binding site enrichment using a discriminative motif analysis (DREME [38]; Fig. 4).

The binding motif “ADTTCGC” was the most significantly enriched out of three motifs enriched among genes overexpressed in the *A. suum* head, and matched MAB-3-like (“Male Abnormal 3”), which has been previously described in *C. elegans* [40]. In *C. elegans*, MAB-3 is required for expression of male-specific genes in sensory neurons of the head, and acts synergistically with LIN-32, a neurogenic bHLH transcription factor [73]. The *A. suum* protein GS_21204 had significant amino acid sequence similarity to the *C. elegans* MAB-3 (E = 2×10^−11^), was annotated with the GO term “sequence-specific DNA binding transcription factor activity” (GO:0003700), and expression for its gene was detected only in the head and pharynx,

Only one binding motif (CATACAYA) was found to be significantly enriched among genes overexpressed in the *A. suum* pharynx. This motif matched the SOX-17 (“SRY-related HMG-box”) transcription factor binding motif previously described in *M. musculus* [40]. While SOX-17 activity has not been studied specifically in nematodes, another SOX protein in *C. elegans* (SOX-1) was found to be one of a small group of transcription factors activated during pharyngeal development [52]. Here, the *M. musculus* SOX-17 protein had high sequence similarity to an *A. suum* protein (GS_07983; E = 4×10^−28^). *GS_07983* was found to be most highly expressed in the pharynx and the head, and was annotated with the KEGG term “SOX1/2/3/14/21 (SOX group B)” (K09267).

The most significant binding motif found among genes overexpressed in the *A. suum* intestine (CTTATCAR) matches the reverse complement binding sequence of ELT-2 (TGATAA), the predominant transcription factor controlling differentiation and function of the *C. elegans* intestine [74] as well as the GATA-like intestine-enriched motif previously reported in *C. elegans* (TCTTATC) [1]. A protein with high sequence similarity to ELT-2 (GS_05212, E = 1×10^−22^) was annotated with the Interpro domain “Zinc finger, NHR/GATA-type” (IPR013088), and its gene was found to be highly expressed in the intestine (as well as in the pharynx).

A *M. musculus* POU2F3 (“pituitary-ocular-Unc-2 family 3”) transcription factor matched the binding motif (TATGCARA) that was the most significantly enriched among genes overexpressed in the *A. suum* testis. This transcription factor is a putative ortholog to the C. elegans gene *CEH-18* (*ZC64.3*) [75], which has been found to be responsible for cell division in gonadal sheath cells [76]. *GS_16028* in *A. suum* was primarily expressed in the testis, shared high protein sequence similarity to CEH-18 (E = 3×10^−34^) and was annotated with the KEGG term “POU domain transcription factor, class 4” (K09366).

A total of 12 predicted binding motifs were enriched among genes overexpressed in the *A. suum* ovary, which may be due to the expression of early-stage developmental genes which are not present in other tissues. This idea is supported by the annotation of the most highly enriched binding motif (GGGGGDK), which matches the ZFP281 (“zinc finger protein 281”) transcription factor binding site in *M. musculus*. The closest ortholog to the *ZFP281* gene in *C. elegans* is *BLMP1* [77], which has very-early embryo developmental activity, but specific genetic targets for this gene have not been previously characterized [78]. In the *A. suum* genome, *GS_10180* was overexpressed only in the ovary, had high protein sequence similarity to ZFP281 (E = 3×10^−16^), and was annotated with a “Zinc finger, C2H2-type” Interpro domain (IPR007087).

In the *A. suum* seminal vesicle, the binding motif (TCGTTMA) matching the *M. musculus* GMEB-1 (Glucocorticoid Modulatory Element Binding protein-1) transcription factor binding motif was the only one that was significantly enriched. There is a known ortholog of *GMEB-1* in *C. elegans* (*C01B12.2*) [75] but its function has not been studied specifically. The *A. suum* protein GS_22365 shares high sequence similarity to GMEB-1 (E = 1×10^−22^). *GS_22365* was highly expressed in the seminal vesicle, and contained a SAND Interpro domain (IPR000770), which is a transcription factor domain also found in GMEB proteins [79].

Finally, the motif “CSCCACW” (which matches the *M. musculus* SMAD3 binding motif) was one of two significantly enriched in the *A. suum* uterus. Although no direct orthologs of this protein have been identified in nematodes, other SMAD transcription factors are known to be involved in a wide range of complex tissue interactions in *C. elegans*, including in many reproductive tissues [80]. GS_00234 in *A. suum* shares high protein sequence similarity with *SMAD3* (E = 4×10^−129^), contained a SMAD Interpro domain (Dwarfin-type; IPR001132), and its gene was overexpressed in the uterus.

These results on binding motif enrichment suggests existence of tissue-specific co-expressed genes that are under similar transcriptional control, and identifies their putative transcription factors in *A. suum*, most of which have putative orthologues that have been previously described in the literature. Several examples provide very promising targets for further study to identify specific mechanisms governing tissue-specific gene expression in adult *A. suum*. The similarities to *C. elegans*, a distant nematode relative to *A. suum*, indicate that findings reported here should have broad applicability to species across the phylum Nematoda. As the annotation of the *A. suum* genome is improved, binding motif enrichment analyses may be improved through the identification of promoters and more accurate sequencing of intergenic regions.

### Constitutively expressed and novel genes

A total of 1,255 genes were constitutively expressed across the tissues. Nineteen GO terms were significantly enriched among these constitutively expressed genes, nearly all of which were related to translational activity, as is expected for eukaryotic housekeeping genes [81] (Supp. Table S2). A total of 4,886 genes were identified as being “novel” based on a lack of annotation from any source. Only 7% of constitutively expressed genes were characterized as novel in this analysis, compared to 23% of all expressed genes (Fig. 5), which is expected since constitutively expressed genes are often conserved and have well-studied biological functions in eukaryotes. Likewise, there were a smaller proportion of novel genes among the gene sets overexpressed in all of the tissues except for the testis (Fig. 5). The significant over-representation of novel genes in the testis (compared to expressed genes not overexpressed in the testis; P<10^−10^) indicates the potential for important and previously undescribed biological functions occurring in the testis of *A. suum*.

### Potential signaling pathways linking reproductive and non-reproductive tissues

Gene co-expression networks, in which genes are represented as nodes and are connected by edges corresponding to their co-expression across a number of samples of gene expression, are a powerful approach for developing hypotheses regarding the functions of both annotated and unannotated genes [82], [83], [84] (including identifying genes related to functions not specifically tested in the source datasets [1], as well as for identifying putative functional modules related to transcriptional activity [46]). Here, sex-independent co-expression networks were constructed (using Cytoscape software V3.0 [45]; Methods) for 4,784 genes overexpressed in male tissues (with 1,387,028 edges; Fig. 6) and for 7,741 genes overexpressed in female tissues (1,188,989 edges; Fig. 7; Methods). In both male and female networks, there are far fewer reproductive to non-reproductive connections in the networks than expected based on the total number of inter-tissue connections. If the network was random, then 60% of the inter-tissue connections should be between reproductive and non-reproductive tissues, but only 4% and 18% of the between-tissue edges were found to connect these tissue types (p<10^−15^ for both networks, binomial distribution test), making the existing reproductive to non-reproductive connections in the network particularly interesting for further study.

The male gene co-expression network automatically arranged in a pattern similar to the body plan of the male *A. suum* worm (Fig. 6A), with the pharynx serving as a bridge between the head and the intestine, and very few connections between the non-reproductive and reproductive tissues. However, a subnetwork of male head, pharynx and intestine genes closely associated with seminal vesicle genes (Fig. 6B) may present a functional link between these tissues. This gene cluster was most significantly enriched for “regulation of transcription, DNA-dependent” (GO:0006355), “sequence-specific DNA binding transcription factor activity” (GO:0003700) and “acetyl-CoA carboxylase activity” (GO:0003989; p = 8×10^−4^, 1×10^−3^ and 4×10^−3^, respectively). At the top-left of this subnetwork is a head-overexpressed gene (*GS_05069*, in red), one of only two head-overexpressed genes not directly connected to the main head-network hub (Fig. 6A). The predicted protein for this gene was matched to the “nicotinic acetylcholine receptor, invertebrate” KEGG category (K05312). Acetylcholine functions as a modulatory neurohormone in *Ascaris lumbricoides* [85], and here *GS_05069* was found to share very high sequence similarity to the *H. contortus* protein Hco-monepantel-1 (E = 4e^−85^), which has been identified as a target for the recently developed anthelmintic drug monepantel (an amino-acetonitrile derivative) [86], [87], [88]. This head-overexpressed gene is only highly correlated with one other gene (*GS_05881*) which was also head-overexpressed and was annotated with a “Nematode fatty acid retinoid binding” Interpro domain (IPR008632), and which shared high protein sequence similarity to FAR-1 in *Onchocerca volvulus* (E = 4e^−72^) [13]. FAR-1 belongs to a family of orthlogous proteins which play important roles in development and reproduction in nematodes [13], [89]. *GS_05881* connects to a subnetwork of intestine-overexpressed genes which are highly correlated with the expression patterns of many seminal vesicle-overexpressed genes, and which are rich with annotations related to transcriptional activity (Fig. 6B). These observations are consistent with the predicted role of FAR-1 in reproduction. Other FAR-1 homologs are a focus of interest in terms of their crucial role in parasitism [90], [91], and have been suggested to be potential targets for new anthelmintics due to their expression on the epidermis, their lack of similarity to any host proteins and their critical function in host environment detection [92], [93]. Here, we present the first evidence that the *A. suum* homologs to Hco-monepantel-1 and FAR-1 (both previously described as anthelminthic drug targets) are co-expressed in *A. suum*, and the networks of genes with similar expression patterns may be used in future research to develop hypotheses about members and functions of the network, or to identify other potential downstream drug targets.

Like in the male network, the female co-expression network (Fig. 7A) arranged in a pattern similar to its body plan layout, with the pharynx bridging the head and intestine and the reproductive tissues largely separated. However, in the female, the subnetwork connecting the head and ovary networks is very dense (Fig. 7B), involving a large set of co-expressed genes. One of the head-overexpressed genes central to this head-ovary bridge network (*GS_05636*) was annotated as an “ecdysone receptor” (K14034). This was the only gene in the current *A. suum* genome annotated to this KEGG category, and is important because in the parasitic nematode *Brugia malayi*, ecdysteroid signalling has been found to play a role in molting and fertility, but the mechanism behind these relationships is unknown [94]. Similar to the story for the male subnetwork, this gene bridges a gap between reproductive and non-reproductive networks (as evidenced by the its first-neighbor co-expression pairs which include both head-overexpressed and ovary-overexpressed genes), and may be an interesting target for further study in order to elucidate signal transduction pathways and design drug targets for eliminating *A. suum* fertility. Although the co-expression networks were segregated by gender here, the pathways described are not necessarily restricted to only one gender.

### Conclusions

The functional enrichment results across different *A. suum* tissues present many confirmations of existing knowledge in nematode tissues, as well as many suggestions of novel functions which are interesting subjects for further study. This analysis indicates that the *A. suum* pharynx may be actively involved in digestive processes and it provided functional descriptions of the *A. suum* seminal vesicle, ovary and uterus, which have not been previously studied in this detail. Constitutively expressed and novel genes were also characterized, and putative tissue-specific transcriptional factors and corresponding binding motifs were deduced stemming from results of the tissue expression analysis, which included the intestine-enriched ELT-2 motif/transcription factor previously described in nematode intestines. Also, the gene co-expression networks constructed here present several possible novel molecular signalling pathways between non-reproductive and reproductive tissues, and provide a resource for quickly identifying genes co-expressed between different tissues. As the *A. suum* genome is better annotated and specific pathways are more carefully identified, additional subnetworks of interest could be identified in these networks.

The analyses in this paper present several approaches for mining data from this rich RNA-seq analysis of 10 different *A. suum* tissues. Hence, the dataset, co-expression relationship and transcriptional regulation that were derived from it provide a valuable resource for studying tissue-specific biological activity in nematodes. In addition, the annotation data, gene expression data and overexpressed gene lists in each tissue (Supp. Table 1; also deposited into www.nematode.net, enabling readers to perform advanced searches) provide valuable resources for building future tissue-specific analyses for helping with drug and vaccine design directed against parasitic nematodes.

## List of Abbreviations

DCPM – Depth of coverage per million reads mapped
GO – Gene Ontology
KEGG – Kyoto Encyclopedia of Genes and Genomes
MF – Molecular Function
BP – Biological Process
CC – Cellular Component

## Accessions

Processed and raw paired-end RNA-seq datasets are deposited at the sequence reads archive (SRA) on the NCBI website (http://www.ncbi.nlm.nih.gov/sra; Accession Numbers SRR85166, SRR85167, SRR851186-SRR851203, SRR851213, SRR851223-SRR851225, SRR851254-SRR851257, SRR851632-SRR851637, SRR851639-SRR851641, SRR851855-SRR851857, SRR869476, SRR851237, SRR851252, SRR851258 and SRR869505). Reads were mapped to the *A. suum* genome assembly produced and described by Jex et al. (Nature, 2011), and all gene names used in this manuscript are consistent with the gene names in that publication.

## Supporting Information

Figure S1 (A) The overlap of genes identified with ≥50% breadth of coverage in whole-worm male *A. suum* samples and the merged male tissue samples (head, pharynx, intestine, testis and seminal vesicle). (B) The overlap of genes identified with ≥50% breadth of coverage in whole-worm female *A. suum* samples and the merged female tissue samples (head, pharynx, intestine, ovary and uterus).
(TIF)

Figure S2 Highly significantly enriched (p≤10^−5^, FDR corrected) Interpro domains among genes overexpressed in each of the non-reproductive tissues.
(TIF)

Figure S3 Highly significantly enriched (p≤10^−5^, FDR corrected) Interpro domains among genes overexpressed in each of the reproductive tissues.
(TIF)

Table S1 *A. suum* gene annotation, expression, and overexpression results.
(XLSX)

Table S2 FUNC Gene Ontology (GO) enrichment significance values for genes overexpressed in each tissue studied.
(XLSX)

Table S3 Tomtom motif-matching output, showing the most enriched binding motifs in each tissue (bottom) and their corresponding best-matching transcription factor binding motif (top).
(XLSX)

Table S4 Base ambiguity code interpretation for the motif enrichment study.
(XLSX)

Table S5 BLAST results for the five *A. suum* MDP genes presented in Tarr and Scott (2004).
(XLSX)
