# Supplementary figures and images for: Genome-Wide Tissue-Specific Gene Expression, Co-expression and Regulation of Co-expressed Genes in Adult Nematode Ascaris suum
Source: PLoS Negl Trop Dis. 2014 Feb 6;8(2):e2678. doi: 10.1371/journal.pntd.0002678 (PMC3916258; doi:10.1371/journal.pntd.0002678)

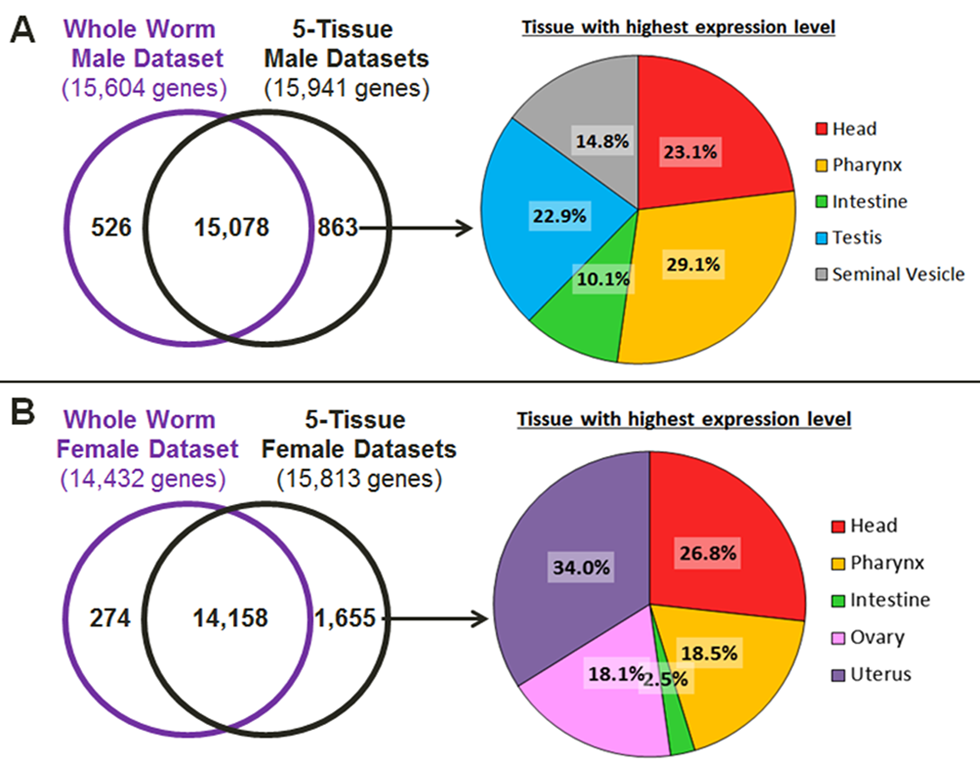

Supplement: Figure S1 — (A) The overlap of genes identified with ≥50% breadth of coverage in whole-worm male A. suum samples and the merged male tissue samples (head, pharynx, intestine, testis and seminal vesicle). (B) The overlap of genes identified with ≥50% breadth of coverage in whole-worm female A. suum samples and the merged female tissue samples (head, pharynx, intestine, ovary and uterus). (TIF) [file pntd.0002678.s001.tif]

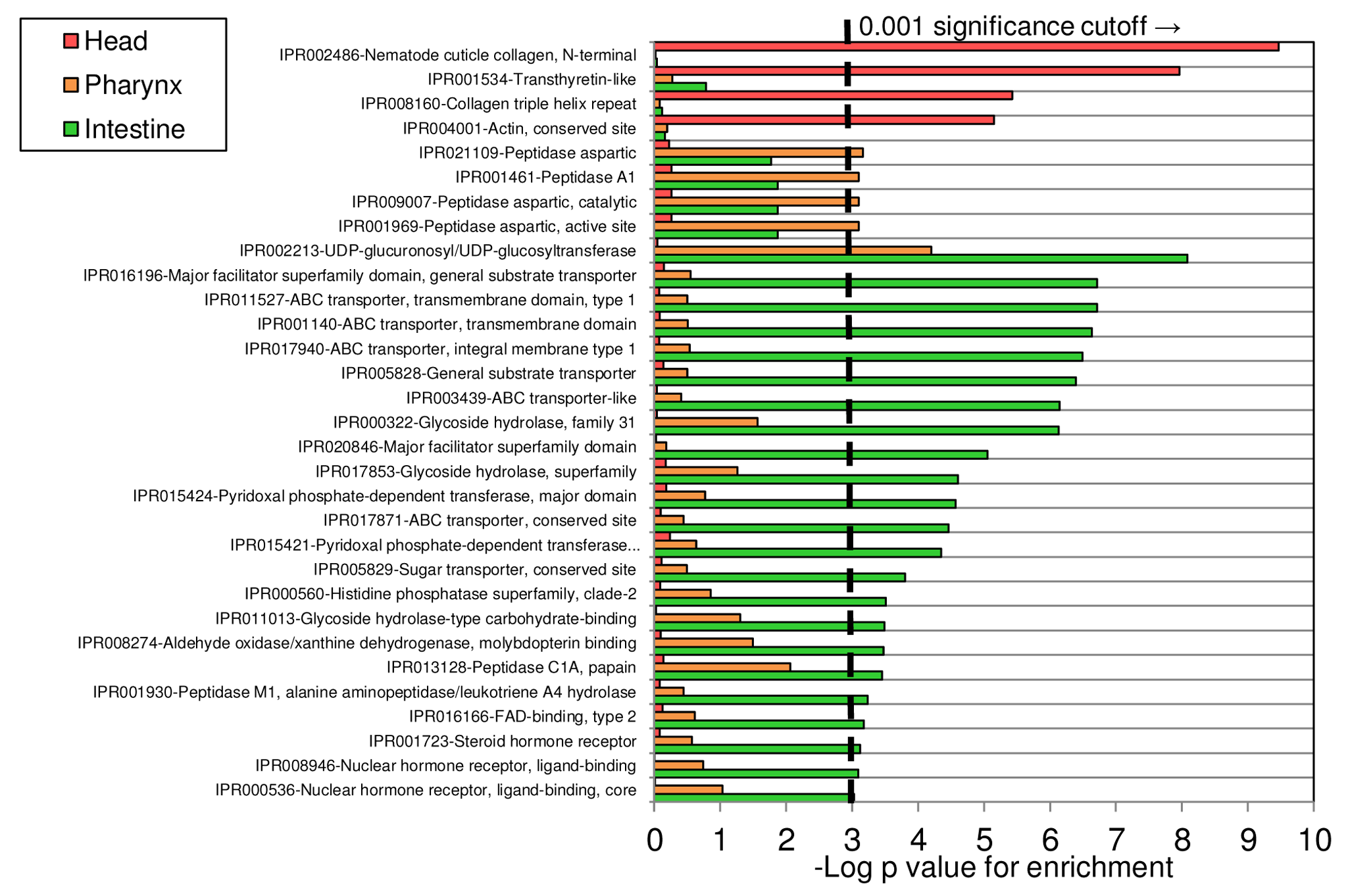

Supplement: Figure S2 — Highly significantly enriched (p≤10−5, FDR corrected) Interpro domains among genes overexpressed in each of the non-reproductive tissues. (TIF) [file pntd.0002678.s002.tif]

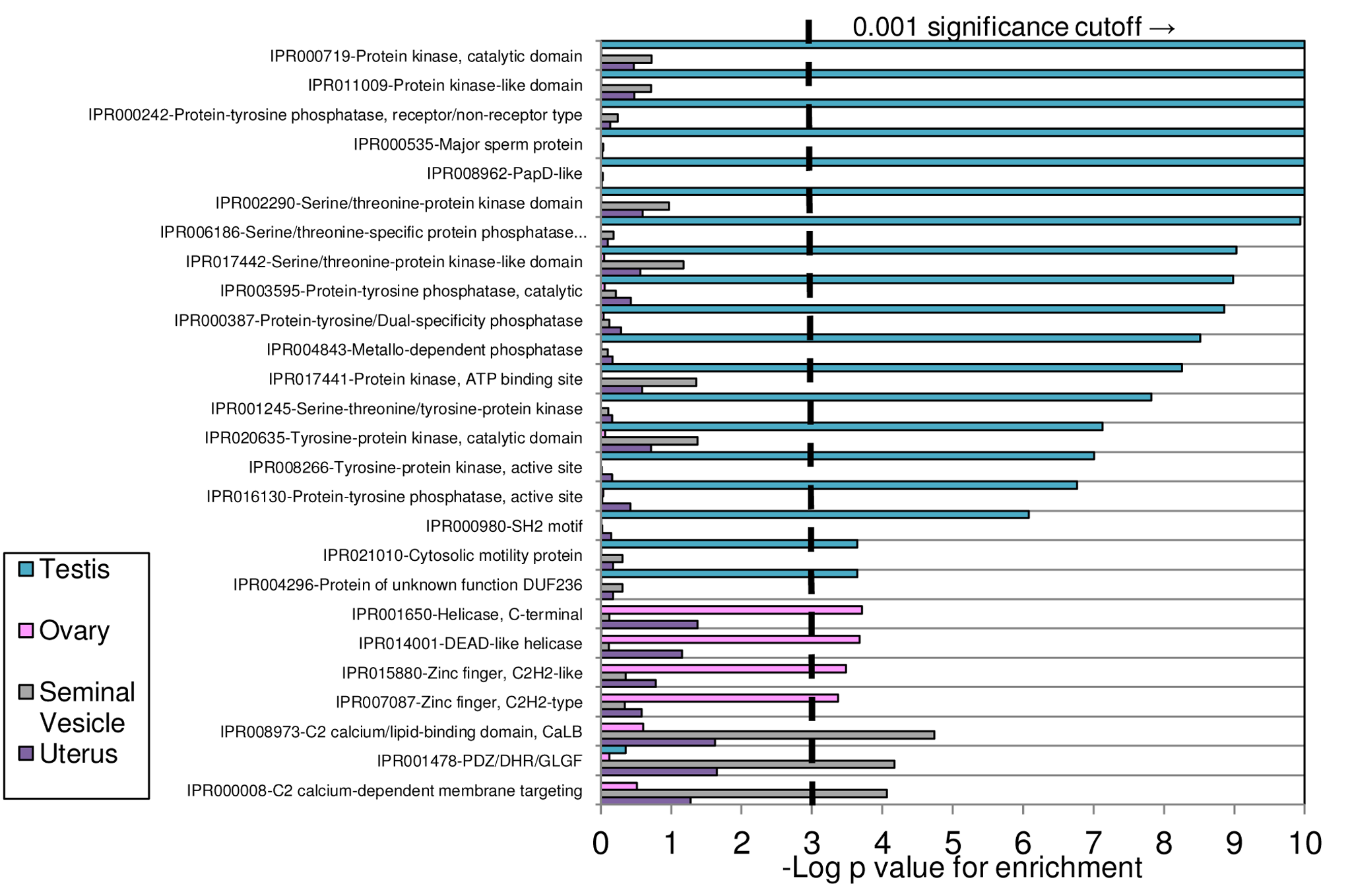

Supplement: Figure S3 — Highly significantly enriched (p≤10−5, FDR corrected) Interpro domains among genes overexpressed in each of the reproductive tissues. (TIF) [file pntd.0002678.s003.tif]
